# Bergen social media engagement and experiences scale (Be-SMEE): A short questionnaire covering important aspects of social media use among adolescents. Development and association with symptoms of depression and anxiety.

**DOI:** 10.12688/f1000research.161622.1

**Published:** 2025-02-21

**Authors:** Jens Christoffer Skogen, Turi Reiten Finserås, Børge Sivertsen, Ian Colman, Amanda Iselin Olesen Andersen, Gunnhild Johnsen Hjetland

**Affiliations:** 1 Center for alcohol and drug research (KORFOR), Stavanger University Hospital, Stavanger, Norway; 2 Centre for Evaluation of Public Health Measures, Norwegian Institute of Public Health, Oslo, Norway; 3 Department of Health Promotion, Norwegian Institute of Public Health, Bergen, Norway; 4 Department of Research and Innovation, Helse Fonna HF, Haugesund, Rogaland, Norway; 5 Centre for Fertility and Health, Norwegian Institute of Public Health, Oslo, Norway; 6 School of Epidemiology and Public Health, University of Ottawa, Ottawa, Canada

**Keywords:** social media, adolescence, mental health, questionnaire

## Abstract

**Background:**

There is a need to go beyond mere measures of time used on social media. Existing tools inadequately capture the multidimensional nature of social media use, leaving a gap for concise yet comprehensive assessment tools.

**Aims:**

This study aimed to develop a short questionnaire addressing three critical dimensions of social media use: self-presentation, negative experiences, and problematic use. The association between these dimensions and symptoms of anxiety and depression was also investigated.

**Methods:**

This study uses two independent datasets of adolescents aged 16+ years in Norway. Using Ant Colony Optimization (ACO) analyses, a pool of 31 social media items was analyzed to investigate factor structure and associations with symptoms of anxiety and depression. For model development, the “LifeOnSoMe”-study was employed (>3,500 participants), and data from a pilot study (~500 participants) was used for external validation.

**Results:**

Based on ACO-analyses, a 20-item six-factor model was identified, encompassing social comparison and self-presentation (five items), and three items for each of the following domains: negative experiences (Negative acts and Unwanted attention from others) and problematic use (Subjective overuse, Social obligations, and Source of concern). Confirmatory factor analyses demonstrated very good to excellent fit in both datasets, and consistent associations between the six different domains and symptoms of anxiety and depression.

**Discussion:**

The suggested 20-item questionnaire provides a robust and succinct tool for evaluating social media’s impact on mental health, offering substantial explanatory power for variance in anxiety and depression symptoms. This can serve as meaningful tool for assessing the potential impact of social media use for mental health and related outcomes.

## Introduction

In the last 15 years, a large volume of research has been focused on the potential link between social media use and mental health outcomes. ^1– 4^ Initially, the primary focus was on the amount of time used on social media, ^1, 5, 6^ but currently a more fine-grained and purposeful focus has been advocated. ^4, 7^ This reflects the growing awareness that the total time spent on social media is a crude and imprecise measure for a host of different exposures and interactions. ^1, 8– 10^ How social media influences mental health likely depends on several interrelated factors, including platform affordances, content, individual characteristics, and broader contextual determinants. ^1, 4, 9^ In line with this, our previous research has focused on more specific aspects of social media use. ^11– 16^ In general, we have focused on three superordinate domains in relation to social media use: self-presentation and social comparison, ^12, 16^ negative experiences, ^14, 15, 17^ and problematic use. ^11^ These domains are frequently highlighted in the literature as important factors when investigating the potential negative impact of social media use on mental health. ^1, 9, 18– 22^ Previous research has shown that these domains are consistently related to mental health and well-being among adolescents, for both boys and girls. ^11– 16^ More negative experiences on social media are for instance related to more symptoms of anxiety and depression, and this association with mental health is also true for higher levels of problematic social media use and aspects of self-presentation.

Given the complexity in gauging what social media use entails, ^7, 13^ a challenge is to be able to capture several different domains succinctly and without lengthy questionnaires. This challenge is not unique to social media use but is a familiar phenomenon in many areas of research. ^23, 24^ In surveys, there is always a pressure on the length of the survey, and care is given to ensure that the respondents are not overloaded or are presented with redundant or irrelevant items. In a recent paper by Twivy and colleagues, they used Ant Colony Optimization to develop a 15-item short-form social media scale for depression in adolescence covering five different domains, “hostility from others”, “hostility towards others”, “social comparison”, “passing time” and “seeking support”. ^22^ The areas covered were all associated with adolescent depression and well-being, but it did not specifically include other potentially important domains such as problematic social media use or unwanted attention from others. To the best of our knowledge, no existing questionnaires on social media use simultaneously address the domains outlined below. The present study aimed to leverage items used previously to establish a concise questionnaire covering six domains of social media use (the original number of items in parentheses):

I. Self-presentation and social comparison (7)
II. Negative experiences:
  ○ Negative acts and exclusion (5)
  ○ Unwanted attention from others (3)
III. Problematic social media use
  ○ Subjective overuse (5)
  ○ Social obligations (8)
  ○ Source of concern (3)

In previous publications these domains have been covered by a total of 31 items. Our aim in this paper was to reduce this number substantially using data from two large samples of Norwegian adolescents, while being able to retain the different domains listed above. After item-reduction, we examined the association between these domains and symptoms of anxiety and depression.

## Methods

This study is based on two independent data sources that utilized similar methodology and survey designs. The main data source is the “LifeOnSoMe”-study, which included more than 3,500 adolescents (aged 16+ years) from Bergen Municipality in Norway. The study covered a range of factors potentially associated with mental health and well-being. Importantly, it also contained a separate section specifically investigating several different aspects of social media use (for more information see below). The second data source originates from the pilot study preceding the “LifeOnSoMe”-study which was completed in Alver Municipality, Norway, and included around 500 adolescents (aged 16+ years). Although there are some slight discrepancies in the survey content across these two data sources, the recruitment procedure and main measure were identical. Importantly, the measures used in the present study are identical across the two surveys. This similarity allows for direct comparison between them. Both surveys were in Norwegian, and age and gender were registered based on self-report. Further information about both data sources, as well as their contextual information, can be found in previous publications. ^12, 14, 16^

### Measures of social media use

In a specific section of the survey, adolescents answered statements about their use, beliefs, experiences, and attitudes toward social media. The items were based on findings from focus group interviews of adolescents (27 adolescents across five groups; for more information, see for instance ^13^). All the statements had five response options, and for the present study, the following domains were of interest (original number of items in parentheses):

Social comparison and self-presentation (e.g. “I spend a lot of time and energy on what I post on social media”, 7 items), Negative acts and exclusion (e.g. “Others say/post bad things about me on social media”, 5 items), Unwanted attention from others (e.g. “I receive unwanted nude photos or sexualized content from others”, 3 items), Subjective overuse (e.g. “I spend too much time on social media”, 5 items), Social obligations (e.g. “I feel that I must respond to all messages, “streaks” and similar things I receive”, 8 items), and Source of concern (e.g. “There is so much happening on social media that I often feel overwhelmed”, 3 items). The specific statements and response options are presented in Table 1.

**Table 1.** Different aspects of social media use. Original statements and response options.

**Question: To what extent are the following statements true for you? (Answer options: Not at all, A little, Somewhat/partly, A lot, Very much)**	
**Social comparison and self-presentation **	1. I spend a lot of time and energy on what I post on social media
	2. It is important for me to get many likes and/or comments on what I post on social media
	3. It is important for me to have many followers on social media
	4. I delete what I post on social media if it does not get enough likes or comments
	5. I retouch photos of myself to look better before posting them on social media
	6. What others post (photos/status updates/stories) makes me feel less content with myself and my own life
	7. The response I get for what I post (photos/status updates/stories) is impacts how I feel
**Subjective overuse**	1. Social media takes away focus from more important things
	2. I am addicted to social media
	3. My parents/guardians think I spend too much time on social media
	4. I spend too much time on social media
	5. I want to reduce the amount of time I spend on social media
**Social obligations**	1. I fear I might miss out on something if I am not on social media
	2. Social media gives me a sense of control or overview of what is going on
	3. I feel that I must like and/or comment on what friends post on social media
	4. I feel that I must respond to all messages, "streaks" and similar things I receive
	5. If I do not respond, like or comment, then it can have negative consequences
	6. If my friends do not like or comment on what I post on social media, I start thinking something is wrong
	7. If I do not participate on social media, I will fall behind
	8. I follow closely what my friends/girlfriend/boyfriend/family does through social media (for example stories, Snap map …)
**Source of concern**	1. I wish we could learn more about how social media affects us
	2. There is so much happening on social media that I often feel overwhelmed
	3. Sometimes I feel like I am being monitored on social media (because what I do/where I am/who I am with, is visible)
**Question: How often does the following happen/do you do the following: (Answer options: Never, Seldom, Sometimes, Often, Very often)**	
**Unwanted attention from others**	1. I get contacted/get unwanted attention from strangers on social media
	2. I receive unwanted nude photos or sexualized content from others
	3. I am asked to send nude photos or sexualized content of myself to others
**Negative acts and exclusion**	1. Others share photos/videos of me against my will
	2. I get negative/rude comments on what I post
	3. I receive unpleasant or hurtful messages through social media
	4. Others say/post bad things about me on social media
	5. I feel excluded from groups/group chats on social media

### Symptoms of anxiety

Symptoms of anxiety were assessed using the General Anxiety Disorder 7 (GAD-7) questionnaire. ^25^ The GAD-7 consists of seven questions about general anxiety symptoms, scored from 1 (“not at all”) to 4 (“almost every day”). It can be used as a continuous measure (total score ranging from 0 to 28) or as a dichotomous variable with a cut-off score of 10 to define case-level anxiety. For this study, the GAD-7 was used as a continuous variable.

### Symptoms of depression

Symptoms of depression were measured using the Short Mood and Feelings Questionnaire (SMFQ ^26^). The SMFQ includes thirteen statements about depressive symptoms, with response options of 0 (“not true”), 1 (“sometimes true”), and 2 (“true”). It can be used as a continuous measure (total score ranging from 0 to 26) or as a dichotomous variable with a cut-off at the 90 ^th^ percentile to define case-level depression. For this study, the SMFQ was used as a continuous variable.

### Statistical analyses

The “LifeOnSoMe”-study proper was designated as the model development dataset, while the pilot study data was designated as the external validation sample. First, descriptive statistics of self-reported age and gender, as well as mental health variables, were presented across the model development and the external validation sample. Frequencies and proportions were estimated for age and gender, while the median and interquartile range were estimated for symptoms of depression and anxiety. Potential differences between the two datasets were estimated using Pearson’s Chi-squared tests for age and gender and Wilcoxon rank sum tests for the mental health variables. Next, item reduction was done using Ant Colony Optimization (ACO). ACO is a metaheuristic algorithm inspired by ants’ foraging behaviour, in this case applied to factor analysis for optimizing measurement scales. ^27^ In our context, artificial ants construct solutions by selecting items, guided by pheromone trails that represent the quality of previous solutions. ^28– 30^ The algorithm iteratively updates these trails, reinforcing paths leading to psychometrically sound scales and factor structure. ^27^ ACO has previously been successfully used to develop short scales, such as for assessing personality ^29^ and the beforementioned short social media scale for depression. ^22^ ACO-analysis was performed using the model development data set. Model fit and factor loadings for the suggested model were estimated across both datasets using confirmatory factor analysis with the Diagonally Weighted Least Squares (DWLS) estimator. DWLS was employed to handle the ordinally scaled items included, providing more accurate parameter estimates and standard errors. Model fit was assessed using the Comparative Fit Index (CFI, good fit: ≥ 0.95), Tucker-Lewis Index (TLI, good fit: ≥ 0.95), Root Mean Square Error of Approximation (RMSEA, good fit ≤ 0.06) and Standardized Root Mean Square Residual (SRMR, good fit ≤ 0.08). Configural and scalar measurement invariance ^31^ were also tested across the two datasets, as well as across gender and age (see Table 5). As per recommendations for ordinal indicators, we bypassed metric invariance testing and directly assessed scalar invariance by simultaneously constraining factor loadings and thresholds. ^32, 33^ This approach is more appropriate and parsimonious for ordinal data, as both loadings and thresholds jointly determine response probabilities. For measurement invariance, we jointly considered ΔCFI ≤ -0.01 and ΔRMSEA ≤ 0.015 as evidence of invariance across groups. Finally, Bayesian linear regression models ^34^ adjusted for age and gender were separately estimated between each of the suggested domains (summed average score for each domain) and symptoms of anxiety and depression as dependent variables across both data sets. The following estimates were obtained from the regression models; the coefficient and the corresponding 95% credible interval and the probability of direction. For the regression models, the dependent variables were standardised (Z-scored; mean of 0 and standard deviation of 1) in each data set. Additionally, the Bayes factor and the error percentage were estimated when comparing an age- and gender-only model (baseline) versus a model that also included the social media domains. Bayes factor estimates the relative evidence for one statistical model over another by comparing their predictive performance. ^35^ Potential differences in regression estimates across the two datasets were investigated in moderation analyses in a combined dataset with a grouping variable term: dependent variable×dataset. Moderation by dataset was considered present when the credible interval of the interaction term did not cross zero. For the development dataset, a total of 3,285 participants had valid responses for all variables of interest and were included in the analytical sample. In comparison, the external validation dataset included 509 participants with valid responses. Missing data was handled by case-wise deletion, with a maximum of 7.7% of the total number of participants excluded in any of the analyses. All analyses were done using R Studio. ^36^ ACO-analyses were performed using the ‘ShortForm’-package, ^37^ while Bayesian linear regression models were computed using ‘rstanarm’ ^38^ and Bayes factor estimates were derived from the ‘BayesFactor’-package. ^39^ Confirmatory factor analyses were done using the ‘lavaan’-package. ^40^ Tables were produced using the packages ‘gtsummary’ ^41^ and ‘flextable’. ^42^

## Results

Table 2 provides descriptive statistics for the model development and the external validation sample. There were some age and gender differences between the two samples, with a slightly higher age (mean age 17.3 vs 17.1 years, p<0.001) and a higher proportion of girls (56% vs 42%, p<0.001) in the former sample compared to the latter. There were no differences in terms of symptoms of anxiety and depression (p-values >0.05). Based on results from the ACO approach, the best fitting model was a 20-item, six-factor model (see Table 3) which included five items for self-presentation, and three items for the rest of the domains (Negative acts, Unwanted attention from others, Subjective overuse, Social obligations, and Source of concern). This constituted a 35% reduction of items compared to the original number of items. Results from the confirmatory factor analysis indicated very good to excellent model fit and satisfactory factor loadings in both samples (see Table 3 and Table 4). Model fit in the model development dataset was CFI: 0.968, TLI: 0.960, RMSEA: 0.058 and SRMR: 0.039, compared to CFI: 0.978, TLI: 0.973, RMSEA: 0.056 and SRMR: 0.055 in the external validation dataset. Measurement invariance testing indicated that the suggested model fits across the age and gender, as well across the two samples (see Table 5). Overall, factor loadings were consistent across both samples, with all standardized factor loadings exceeding 0.5.

**Table 2.** Descriptive statistics of demographic and mental health variables across method development and external validation sample.

Variables	Method development N = 3,285 ^1^	External validation N = 509 ^1^	p-value ^2^
Age			<0.001
16	600 (18%)	163 (34%)	
17	1,573 (48%)	178 (37%)	
18	901 (27%)	88 (18%)	
19+	211 (6.4%)	48 (10%)	
Gender			<0.001
Boys	1,433 (44%)	296 (58%)	
Girls	1,852 (56%)	213 (42%)	
SMFQ - depression	5 (2, 10)	5 (2, 10)	0.2
GAD - anxiety	5.0 (2.0, 8.0)	5.0 (2.0, 8.0)	0.6

^1^ n (%); Median (Q1, Q3).

^2^ Pearson's Chi-squared test; Wilcoxon rank sum test.

**Table 3.** Factor loadings across method development and external validation sample. Short version.

Factor	Item	Model development		External validation	
		Std. Loading	p-value	Std. Loading	p-value
**Self-presentation and social comparison**	1. I spend a lot of time and energy on what I post on social media	0.69	<0.001	0.68	<0.001
	2. I delete what I post on social media if it does not get enough likes or comments	0.73	<0.001	0.74	<0.001
	3. I retouch photos of myself to look better before posting them on social media	0.60	<0.001	0.60	<0.001
	4. What others post (photos/status updates/stories) makes me feel less content with myself and my own life	0.79	<0.001	0.80	<0.001
	5. The response I get for what I post (photos/status updates/stories) is impacts how I feel	0.85	<0.001	0.89	<0.001
**Negative acts**	6. Others say/post bad things about me on social media	0.89	<0.001	0.95	<0.001
	7. I get negative/rude comments on what I post	0.93	<0.001	0.96	<0.001
	8. I receive unpleasant or hurtful messages through social media	0.92	<0.001	0.90	<0.001
**Unwanted attention**	9. I receive unwanted nude photos or sexualized content from others	0.87	<0.001	0.82	<0.001
	10. I am asked to send nude photos or sexualized content of myself to others	0.92	<0.001	0.93	<0.001
	11. I get contacted/get unwanted attention from strangers on social media	0.80	<0.001	0.81	<0.001
**Subjective overuse**	12. I want to reduce the amount of time I spend on social media	0.77	<0.001	0.78	<0.001
	13. I spend too much time on social media	0.86	<0.001	0.88	<0.001
	14. My parents/guardians think I spend too much time on social media	0.69	<0.001	0.71	<0.001
**Social obligations**	15. If my friends do not like or comment on what I post on social media, I start thinking something is wrong	0.87	<0.001	0.90	<0.001
	16. feel that I must respond to all messages, "streaks" and similar things I receive	0.54	<0.001	0.63	<0.001
	17. If I do not respond, like or comment, then it can have negative consequences	0.76	<0.001	0.80	<0.001
**Source of concern**	18. There is so much happening on social media that I often feel overwhelmed	0.81	<0.001	0.76	<0.001
	19. Sometimes I feel like I am being monitored on social media (because what I do/where I am/who I am with, is visible)	0.67	<0.001	0.71	<0.001
	20. I wish we could learn more about how social media affects us	0.56	<0.001	0.58	<0.001

**Table 4.** Model fit indices across method development and external validation sample.

Measure	Values (Model development)	Values (External validation)
Chi-square	1754.104	379.918
Degrees of freedom	155.000	155.000
CFI	0.968	0.978
TLI	0.960	0.973
RMSEA	0.058	0.056
SRMR	0.039	0.055

Note: Comparative Fit Index (CFI, good fit: ≥ 0.95), Tucker-Lewis Index (TLI, good fit: ≥ 0.95), Root Mean Square Error of Approximation (RMSEA, good fit ≤ 0.06) and Standardized Root Mean Square Residual (SRMR, good fit ≤ 0.08).

**Table 5.** Model fit indices for measurement invariance testing.

Model	CFI	RMSEA	ΔCFI	ΔRMSEA
*Across model development and external validation*				
Configural	0.971	0.056	-	-
Scalar	0.974	0.048	0.003	-0.008
*Across gender*				
Configural	0.962	0.059	-	-
Scalar	0.960	0.055	-0.002	-0.004
*Across age groups*				
Configural	0.970	0.058	-	-
Scalar	0.971	0.051	0.001	-0.007

Note: Comparative Fit Index (CFI, good fit: ≥ 0.95) and Root Mean Square Error of Approximation (RMSEA, good fit ≤ 0.06). For measurement invariance, we considered ΔCFI ≤ -0.01 and ΔRMSEA ≤ 0.015 as evidence of invariance across groups.

With respect to symptoms of anxiety and depression (see Table 6), all the suggested factors were reliably and positively associated with increased symptoms across the two samples. For all results, the probability of direction strongly supported a positive association. Furthermore, the Bayes factor provided strong-to-extreme evidence favoring a model including social media use domains over an age- and gender-only model. ^35^ In general, point estimates were similar across the two datasets. However, there was one notable exception: the association between subjective overuse and symptoms of depression was slightly stronger in the external validation dataset. The full questionnaire accounted for approximately one-quarter (26-28%) of the variability in symptoms of depression and anxiety in both datasets.

**Table 6.** Bayesian linear regression models across model development and external validation sample. Age- and gender-adjusted.

		Model development					External validation				
Dependent variable	Parameter	Median	95% CI	Probability of direction	Bayes factor	Error percentage	Median	95% CI	Probability of direction	Bayes factor	Error percentage
Symptoms of anxiety (GAD)	Self-presentation	0.40	(0.36, 0.45)	100	>100	0.01	0.42	(0.31, 0.55)	100	>100	0.01
	Negative acts	0.36	(0.31, 0.42)	100	>100	0.00	0.34	(0.21, 0.49)	100	>100	0.01
	Unwanted attention	0.28	(0.24, 0.32)	100	>100	0.00	0.28	(0.18, 0.39)	100	>100	0.00
	Subjective overuse	0.12	(0.08, 0.15)	100	>100	0.01	0.18	(0.09, 0.27)	100	>30, ≤100	0.00
	Social obligations	0.19	(0.15, 0.23)	100	>100	0.00	0.19	(0.10, 0.27)	100	>100	0.00
	Source of concern	0.26	(0.22, 0.29)	100	>100	0.00	0.31	(0.20, 0.41)	100	>100	0.00
Symptoms of depression (SMFQ)	Self-presentation	0.43	(0.39, 0.48)	100	>100	0.01	0.47	(0.36, 0.58)	100	>100	0.01
	Negative acts	0.44	(0.38, 0.49)	100	>100	0.00	0.41	(0.28, 0.54)	100	>100	0.00
	Unwanted attention	0.29	(0.25, 0.33)	100	>100	0.00	0.27	(0.17, 0.37)	100	>100	0.00
	Subjective overuse	0.11	(0.07, 0.14)	100	>100	0.01	0.22	(0.13, 0.31)	100	>100	0.00
	Social obligations	0.21	(0.18, 0.25)	100	>100	0.00	0.22	(0.13, 0.30)	100	>100	0.00
	Source of concern	0.22	(0.18, 0.26)	100	>100	0.00	0.31	(0.21, 0.41)	100	>100	0.00

Note: Dependent variables are standardised (Z-scored) in each sample. Estimates thus represent average change in the dependent variable expressed as standard deviations for each unit increase in the independent variable. CI: Credible Interval. A probability of direction of 100 indicates that 100% of the posterior distribution of the parameter is on one side of zero. A Bayes factor greater than 100 indicates extreme evidence for the model including the social media domain versus the model with only age and gender. A Bayes factor greater than 30 and not over 100 indicates very strong evidence for the model including the social media domain versus the model with only age and gender.

## Discussion

Our analyses identified a concise 20-item questionnaire encompassing six domains of social media use. Model fit indices and measures of reliability consistently demonstrated very good to excellent fit between the suggested model and the data in both datasets. Furthermore, all six domains were consistently associated with symptoms of anxiety and depression across both data sets. These findings align with previous studies using the original longer versions of the suggested domains within the same datasets. ^11– 16^ The full questionnaire also accounted for a substantial proportion of the variance in symptoms of anxiety and depression across both data sets. The fact that results from the method development dataset were consistently confirmed in the dataset used for external validation indicates that the proposed questionnaire is robust and relevant across cohorts. This consistency was also reiterated when testing for measurement invariance across the two datasets. As all of the items are derived from focus group interviews with adolescents, it is likely that they are experientially relevant when considering social media and potential impact. ^43^

Furthermore, the proposed 20-item questionnaire addresses several limitations inherent in previous instruments, such as platform-dependency or conceptual overlap. ^44^ Specifically, in relation to problematic social media use, previous scales have been criticized for also including symptoms of mental health problems, thus inflating the apparent relationship between the two constructs. ^45^ The questionnaire also includes more common negative experiences which have gained increasing interest lately. ^20^ Overall, our findings suggest that the proposed questionnaire is a useful and succinct tool for assessing critical aspects of social media use among adolescents.

### Implications

The suggested questionnaire could potentially have public health relevance as a reliable and efficient tool for assessing social media use and its association with mental health among adolescents. The questionnaire can potentially help identify adolescents at risk of anxiety and depression related to social media use, enabling early intervention and support. The relatively high level of explained variance suggests that the questionnaire captures a substantial portion of the social media aspects related to depression and anxiety symptoms, highlighting its potential relevance in understanding these mental health issues in a modern technology-oriented society.

Public health professionals can use scores from the questionnaire to design targeted interventions aimed at addressing potential links between social media use and mental health outcomes in adolescents. Findings from research employing the questionnaire can inform policymakers about the relationship between social media and adolescent mental health, helping to shape regulations and guidelines that promote healthier social media habits. For research purposes, the suggested questionnaire provides a comprehensive framework for understanding how different aspects of social media use relate to important outcomes such as mental health problems and well-being. By exploring the associations between specific domains of social media use and mental health outcomes, researchers can gain deeper insights into the mechanisms underlying these relationships. It can also help ongoing research to monitor trends in social media use and the effects of social media use, and this knowledge could potentially be used in intervention studies, helping to adapt public health strategies over time. Ultimately, the proposed questionnaire has the potential to enhance understanding of mental health challenges associated with social media use, contributing to improved health outcomes for adolescents.

### Strengths and limitations

The present study has several notable strengths. Firstly, by using the ACO approach to reduce the number of items, we identified a relatively short questionnaire that effectively captures six different domains related to social media use. ^46, 47^ Secondly, we leveraged two independent yet comparable datasets, enabling robust external validation of the questionnaire in terms of both factor structure and its relationship with symptoms of anxiety and depression. This also meant that we were able to do measurement invariance testing across the two datasets. Although the practical relevance of (especially higher order) measurement non-invariance has been debated, ^31, 48, 49^ it is a strength that we were able to test for configural and scalar invariance in two independent samples. It is also a strength in itself that we were able to investigate the convergent validity and relevance of the suggested domains against frequently used and validated scales focusing on symptoms of depression and anxiety. ^25, 26^ Thirdly, the items included in the suggested questionnaire are less platform-dependent and likely more robust to changes in functionality and mere usage patterns on social media. This is especially important as social media is often thought of as a moving target in research, ^50^ and we believe that the suggested items are less prone to being outdated by changes to the underlying technology or user interface.

However, several limitations should be acknowledged. Firstly, although the suggested questionnaire covers key domains related to social media use, it does not encompass all relevant dimensions. ^9, 22^ Social media use and aspects of social media is complex and multidimensional, and depending on the focus of interest, other domains may be more or less important to assess than those included here. For example, the questionnaire primarily captures risk-related aspects, leaving out positive dimensions of social media use that might buffer against mental health problems. However, we believe that the suggested questionnaire covers six domains that are likely to play a crucial role in our understanding of how social media use may be a health determinant, especially for factors related to mental health and well-being. Secondly, the study is based on cross-sectional data, which limits our ability to infer causality or temporality relationships. Future research should investigate changes over time for the suggested domains and longitudinal associations with for instance mental health. Thirdly, as both datasets rely on data collected from upper secondary schools, the age-range is quite limited (range 16-21 years). Future research should investigate how well the suggested questionnaire performs in both older and younger cohorts. Lastly, our study population was geographically restricted to Vestland County, Norway, and both datasets were collected in a Norwegian context. Future research should assess the questionnaire’s relevance, psychometric properties, and cultural adaptability in diverse populations and linguistic contexts.

## Conclusions

There is a pressing need for tools that go beyond measuring mere time spent on social media. As far as we know, there is a paucity in comprehensive yet succinct assessment tools for different aspects of social media use. This study presents a proposed 20-item questionnaire that captures six important aspects of social media use. Our findings indicate that the proposed questionnaire is a valuable tool for assessing the potential impact of social media use on mental health and related outcomes. We believe it represents a meaningful contribution to the research field. Future research should explore the questionnaires utility in other contexts, and populations, as well as its applicability to outcomes beyond those investigated here.

## Authors’ contributions

Conceptualization, JCS and GJH; methodology, JCS, GJH and TRF; formal analysis, JCS; investigation, GJH, TRF, AIOA and JCS; writing—original draft preparation, JCS; writing—review and editing, GJH, TRF, BS, IC, AIOA, and JCS; project administration, JCS. All authors have read and agreed to the published version of the manuscript.

## Ethics approval and consent to participate

The data collections were approved by the Regional Ethics Committee (REK) in Norway (reference number REK #65611, date of approval 16.12.2019) and was conducted in compliance with the principles outlined in the Helsinki Declaration. All participants were provided with information about the study’s overall objectives, both digitally and through communication with their teacher, and they provided electronic informed consent when participating. It was also made clear that participants had the option to withdraw from the study at any time. Additionally, all individuals invited to participate were at least 16 years old, granting them the legal capacity to independently provide consent; however, parents or guardians were also informed about the study.

## Compliance with reporting standards

This study adheres to the Strengthening the Reporting of Observational Studies in Epidemiology (STROBE) guidelines to ensure transparent and comprehensive reporting of observational research.

## Consent for publication

Not applicable.

## Data Availability

The datasets analysed during the current study are not publicly available, as they contain sensitive information, and the ethical approval of the study and GDPR preclude public access to these datasets. Requests to access these datasets should be directed to JCS, jens.christoffer.skogen@fhi.no. Access to data can be given under the terms of the ethical approval and in accordance with GDPR. Any individual requesting access to the data must be formally added as a member of the project group, as per the ethical approval. This is done through application from the project leader. Access will only be granted if the request aligns with the terms of the ethical approval, complies with GDPR, and includes a detailed description of the intended use of the data.
