# Supplementary material for: Bergen social media engagement and experiences scale (Be-SMEE): A short questionnaire covering important experiences and perceptions of social media use among adolescents. Development and association with symptoms of depression and anxiety
Source: F1000Res. 2025 Sep 1;14:228. Originally published 2025 Feb 21. [Version 3] doi: 10.12688/f1000research.161622.3 (PMC12423622; doi:10.12688/f1000research.161622.3)
Supplement: Peer review history

# Reviewer report: Reviewer response for version 3

**DOI:** 10.5256/f1000research.187223.r410635

**Published:** 2025-09-10

**Authors:** Christopher Ferguson

**Affiliations:** 1 Department of Psychology, Stetson University, DeLand, FL, USA

I think this version of the paper is much better.  I have no further comments.

Is the work clearly and accurately presented and does it cite the current literature?

Partly

If applicable, is the statistical analysis and its interpretation appropriate?

Partly

Are all the source data underlying the results available to ensure full reproducibility?

No

Is the study design appropriate and is the work technically sound?

No

Are the conclusions drawn adequately supported by the results?

No

Are sufficient details of methods and analysis provided to allow replication by others?

Partly

Reviewer Expertise:

Youth and technology including social media

I confirm that I have read this submission and believe that I have an appropriate level of expertise to confirm that it is of an acceptable scientific standard.

# Reviewer report: Reviewer response for version 2

**DOI:** 10.5256/f1000research.184396.r397478

**Published:** 2025-08-18

**Authors:** Christopher Ferguson

**Affiliations:** 1 Department of Psychology, Stetson University, DeLand, FL, USA

I appreciate this revised version of the manuscript.  I think it is much improved.  With a few more edits to language I think this could likely be accepted.

 For one...you must remain open to the null hypothesis simply being true.  For instance, I would edit the sentence, "How social media influences mental health likely depends on several interrelated factors..." to instead read, "Whether social media influences mental health may depend on several interrelated factors...although it is possible even these may prove trivial or null."

 Same here, I'd temper the language, "Previous research has shown that these domains are more consistently related ..." I think you're going to at least need to add "...although once again, effects tend to be small and some null studies are also seen."  for instance, social comparison has NOT been found to be a consistent predictor of outcomes related to social media (even some girls see *improved* body satisfaction with social media use, for isntance).  I think I would deephasize social comparison in that, and other sentences following, given lack of clarity in that area (which extends to studies of social comparison outside of social media into more traditional media as well).

 I think too the authors are going to acknowledge somewhere in here that these correlations, small though they are (and the authors do need to acknolwedge that effect size), may have more to do with different scales tapping into "general distress" rather than anything really having to do with social media (again, the degree to which time spent on social media is such a poor predictor is one such key).

 "Measures of social media use" may be confused for time spent on social media.  I wonder if there's a better description for what this is actually measuring, maybe something like "Perceptions of social media" or "experiences on social media".  This was a problem with a lot of the body image literature where *internal* measures of personality (i.e., a tendency to compare oneself with others) was confused with media exposure.  I think this scale is measuring *perceptions* of experiences on social media...which, we should note, may or may not be accurate (see Sheeringa's recent work on adolescent self-report, which is highly unreliable).  However, one's perception of something still can be important to note...two people may have very different perspectives on the same external experience...that says very little about the external experience per se, but could still be diagnostic for the individual.  I think it would be very helpful for the authors to make clear so their scale is not misinterpreted.

 Similarly, in the discussion I suspect "...six domains of social media use" should be reframed as "six domains of perceptions of social media."

 Edit "pressing need" to just "need"...emotional adjectives are the devil's tool in science.

 For table 6 are the estimates standardized coefficients?  You might want to make a note that, since these don't control for any control variables, these shouldn't be used as robust estimators of unique predictive value of these domains, they are simply for the purposes of basic validation.  

 Also related to Table 6, I believe Anastazi generally recommended correlations of .3 to.4 for validation studies (I certainly concur with that).  With that in mind, some of the correlations may be on the weaker end, which should be noted.  In particular, the coefficients for "subjective overuse" are likely too weak to constitute evidence for validity, and this should be noted.  that may actually, in fact, go back to my previous comments about some of the questions likely being rather weak (which is not the authors' fault, there are larger controversies about some of these diagnostic issues).

 Once again, go through the manuscript and edit "social media use" to be something more accurate to what this is "perceptions of social media" or something like that.  This is not the sort of thing that reliably measures any kind of external "use" but rather is an internal personality inventory.

Is the work clearly and accurately presented and does it cite the current literature?

Partly

If applicable, is the statistical analysis and its interpretation appropriate?

Partly

Are all the source data underlying the results available to ensure full reproducibility?

No

Is the study design appropriate and is the work technically sound?

No

Are the conclusions drawn adequately supported by the results?

No

Are sufficient details of methods and analysis provided to allow replication by others?

Partly

Reviewer Expertise:

Youth and technology including social media

I confirm that I have read this submission and believe that I have an appropriate level of expertise to confirm that it is of an acceptable scientific standard, however I have significant reservations, as outlined above.

# Reviewer report: Reviewer response for version 2

**DOI:** 10.5256/f1000research.184396.r397479

**Published:** 2025-08-09

**Authors:** Vincent Paquin

**Affiliations:** 1 Maastricht University, Maastricht, The Netherlands

The authors have adequately responded to my previous review and I have no further comments to make.

Is the work clearly and accurately presented and does it cite the current literature?

Partly

If applicable, is the statistical analysis and its interpretation appropriate?

Yes

Are all the source data underlying the results available to ensure full reproducibility?

Partly

Is the study design appropriate and is the work technically sound?

Yes

Are the conclusions drawn adequately supported by the results?

Partly

Are sufficient details of methods and analysis provided to allow replication by others?

Yes

Reviewer Expertise:

Psychiatry, digital culture

I confirm that I have read this submission and believe that I have an appropriate level of expertise to confirm that it is of an acceptable scientific standard.

# Reviewer report: Reviewer response for version 1

**DOI:** 10.5256/f1000research.177674.r391575

**Published:** 2025-06-16

**Authors:** Vincent Paquin

**Affiliations:** 1 Maastricht University, Maastricht, The Netherlands

Overall, this study makes a helpful contribution to the literature by proposing a scale that evaluates specific domains of social media use among adolescents, with validation of the factor structure in an independent sample. A notable strength is that the items were developed following focus groups with adolescents. Revisions to the literature review and a more prudent interpretation of the findings would strengthen this article.

 - I agree with the first reviewer’s recommendation to further balance the literature review. There is a fairly consistent association between problematic use and depression-anxiety in the literature, but there is controversy on whether problematic use is a valid concept in the first place, and whether its association with mental health really reflects causal effects.

 - The authors’ approach to focus on specific domains of social media use, such as self-presentation and negative experiences, is relevant and mirrors qualitative research conducted with youth by this group of researchers and others.

 - Although I agree with the first reviewer that we should avoid pathologizing statements such as “I spend a lot of time and energy on what I post on social media”, this item may nonetheless may a valuable indicator of the adolescent’s concern for self-presentation on social media, as reflected in the factor label proposed by the authors.

 - I suggest that the authors clarify the rationale for examining associations with depression and anxiety. Are these associations interpreted as indicators of the validity of the scale (in which case, a more balanced literature review, indicating the inconsistent and often weak associations of social media with mental health would be essential), or are the associations exploratory, with the goal of providing an initial description of the psychological correlates of the scale?

 - I share the first reviewer’s scepticism about interpreting the % of variance in depression/anxiety explained by the scales, especially considering the cross-sectional nature of the data and the potential for common method bias. In my opinion, the associations with depression and anxiety should be interpreted very conservatively.

 - I would be more prudent on proposing public health implications of the scale for the reasons indicated above.

 - It may be helpful to acknowledge other existing scales that address specific domains of social media use (e.g., refer to 1, 2 and 3 ).

Is the work clearly and accurately presented and does it cite the current literature?

Partly

If applicable, is the statistical analysis and its interpretation appropriate?

Yes

Are all the source data underlying the results available to ensure full reproducibility?

Partly

Is the study design appropriate and is the work technically sound?

Yes

Are the conclusions drawn adequately supported by the results?

Partly

Are sufficient details of methods and analysis provided to allow replication by others?

Yes

Reviewer Expertise:

Psychiatry, digital culture

I confirm that I have read this submission and believe that I have an appropriate level of expertise to confirm that it is of an acceptable scientific standard, however I have significant reservations, as outlined above.

## References

[1] : Development and initial evaluation of a multidimensional digital stress scale. *Psychological Assessment* .2021;33(3) : 10.1037/pas0000979 230-242 10.1037/pas0000979 33507798

[2] : Social media mindsets: a new approach to understanding social media use and psychological well-being. *Journal of Computer-Mediated Communication* .2023;29(1) : 10.1093/jcmc/zmad048 10.1093/jcmc/zmad048

[3] : Measuring Interests Not Minutes: Development and Validation of the Adolescents’ Digital Technology Interactions and Importance Scale (ADTI). *Journal of Medical Internet Research* .2020;22(2) : 10.2196/16736 10.2196/16736 PMC705583232049068

# Response

**Published:** 2025-07-01

**Authors:** Jens Christoffer Skogen

**Affiliations:** Department of Health Promotion, Norwegian Institute of Public Health, Bergen, Vestland Fylkeskommune, Norway

Reviewer #2: Vincent Paquin; **Approved with reservations.**

 Overall, this study makes a helpful contribution to the literature by proposing a scale that evaluates specific domains of social media use among adolescents, with validation of the factor structure in an independent sample. A notable strength is that the items were developed following focus groups with adolescents. Revisions to the literature review and a more prudent interpretation of the findings would strengthen this article.

**Response: We agree with the reviewer and have revised these parts of the manuscript accordingly (see below).**

 - I agree with the first reviewer’s recommendation to further balance the literature review. There is a fairly consistent association between problematic use and depression-anxiety in the literature, but there is controversy on whether problematic use is a valid concept in the first place, and whether its association with mental health really reflects causal effects.

**Response: We agree that measures and conceptualizations of other aspects of social media use also have it challenges, perhaps especially for “problematic social media use” and “social media addiction”. We do, however, believe that such an approach holds more promise than focusing on time spent on social media (whether is self-report or not log-based). We have tried to nuance the presentation of this section a little more in the first paragraph of the revised manuscript to also show the potential challenges with these other measures. We hope the changes made are in line with the reviewers’ suggestions. We have now added this in the introduction:**

***“These other, more nuanced, measures of social media use are not without their own issues, however. The conceptualisation of problematic social media use, have for instance been based on several different theoretical frameworks (usually with behavioural addiction as a vantage point) and lack of focus on underlying mechanisms, making it difficult to compare results across studies, and challenging legitimacy of findings. Despite challenges in conceptualization, more specific measures of social media use remain a more promising research avenue than mere simple time-based metrics.***

***In line with this effort to move beyond time-based metrics, several different scales have been developed in recent years. These scales typically focus on more specific aspects of social media use, such as digital stress, problematic social media use, social media engagement, digital habits, passive-or-active use, perceived social support or feelings about social media. The diversity of these scales reflects the complex, multifaceted nature of social media as a phenomenon, but also introduces challenges for research synthesis and practical application.”***

 - The authors’ approach to focus on specific domains of social media use, such as self-presentation and negative experiences, is relevant and mirrors qualitative research conducted with youth by this group of researchers and others.

**Response: Thank you.**

 - Although I agree with the first reviewer that we should avoid pathologizing statements such as “I spend a lot of time and energy on what I post on social media”, this item may nonetheless may a valuable indicator of the adolescent’s concern for self-presentation on social media, as reflected in the factor label proposed by the authors.

**Response: We have tried to describe a little more detail about the context of the study what the framing of the invitation letter was as we hope this may help to alleviate some of the concerns regarding the different statements. Specifially we have now added more information about the framing of the study from the invitation letter:**

***“The invitation letter to both the pilot and the “LifeOnSoMe”-study described the project as an initiative to better understand social media as a social arena for adolescents, which could hold benefits as well as challenges. It also explicitly informed participants that the study aimed to explore both positive and negative associations between social media use and young people’s mental health and well-being.”***

 - I suggest that the authors clarify the rationale for examining associations with depression and anxiety. Are these associations interpreted as indicators of the validity of the scale (in which case, a more balanced literature review, indicating the inconsistent and often weak associations of social media with mental health would be essential), or are the associations exploratory, with the goal of providing an initial description of the psychological correlates of the scale?

**Response: We see that this part of aims was unclear. It is indeed to provide an initial exploratory analysis of the potential psychological correlates of the shortened scale. The aims have now been changed to:**

**“In previous publications these domains have been covered by a total of 31 items. Our aim in this paper was to reduce this number substantially using data from two large samples of Norwegian adolescents, while being able to retain the different domains listed above. After item-reduction, we aimed to conduct an initial exploratory analysis of the psychological correlates of the scale, specifically describing associations between the scale domains and symptoms of anxiety and depression among adolescents.”**

**We hope this makes our rationale more explicit. While a detailed rationale for the potential links between each domain and mental health outcomes would be too extensive for our introduction, we refer to previous publications—both from our research and others—that provide more specific explanations and discuss underlying mechanisms. Many of these publications are referenced in the revised introduction.**

 - I share the first reviewer’s scepticism about interpreting the % of variance in depression/anxiety explained by the scales, especially considering the cross-sectional nature of the data and the potential for common method bias. In my opinion, the associations with depression and anxiety should be interpreted very conservatively.

**Response: The interpretation of the explained variance has now been removed from the revised discussion and the abstract Additionally, we now also provide more information about the strength of the different associations across the different domains - both in the results and the discussion section in the revised manuscript.**

 - I would be more prudent on proposing public health implications of the scale for the reasons indicated above.

**Response: We agree and see that we have been overzealous in this section of the manuscript. In the revised version, this section has been extensively shortened and focus on the scale’s potential usefulness in research.**

 - It may be helpful to acknowledge other existing scales that address specific domains of social media use (e.g., refer to 1, 2 and 3 ).

**Response: We agree, and the revised manuscript now provides more information about other measure of more specific aspects of social media use:**

***“In line with this effort to move beyond time-based metrics, several different scales have been developed in recent years. These scales typically focus on more specific aspects of social media use, such as digital stress, problematic social media use, social media engagement, digital habits, passive-or-active use, perceived social support or feelings about social media. The diversity of these scales reflects the complex, multifaceted nature of social media as a phenomenon, but also introduces challenges for research synthesis and practical application.”***

# Reviewer report: Reviewer response for version 1

**DOI:** 10.5256/f1000research.177674.r368605

**Published:** 2025-03-04

**Authors:** Christopher Ferguson

**Affiliations:** 1 Department of Psychology, Stetson University, DeLand, FL, USA

I appreciated the authors efforts to develop a scale that better assesses youth social media use.  However, I have some observations which reduce my enthusiasm.

 First, the authors need to better balance their lit review, particularly they need to cite null studies related to social media use and mental health to make clear time spent is a poor predictor (see for example, recent meta-analyses of Ferguson, Kaye, Branley-Bell & Markey, in press with Professional Psychology: Research and Practice (2025 [Ref-1]) or Yang and Feng, 2024 in Heliyon).  I believe the authors overstate how “consistently related” specific subdomains are as well.  For instance, the concept of problematic social media use remains highly controversial and citing it as beyond controversy reduces my confidence in the authors’ objectivity.

 One issue I worried about was the fairly obvious demand characteristics of the current study which could create false correlations and unwarranted confidence in the survey.  For future studies, including distractor questionnaires may help.

 Some items did not impress me as helpful and may result in overpathologizing social media use (e.g. “I spend a lot of time and energy on what I post on social media”, even “I spend too much time on social media” which many teens may simply because they are constantly told this by parents, teachers, news media, etc., so they think it’s the correct response).  Others such as “My parents/guardians think I spend too much time on social media”…every parent has thought this about every form of media for centuries…it’s just not diagnostic. 

 It occurs to me that single-responder bias may also be a source of elevated correlations. 

 In the regression tables, is “median” the median correlation?  I apologize if I missed that explanation somewhere.  I just wasn’t quite sure what “median” referred to.

 Some validity coefficients are on lower end.  Usually validity correlations in the .3 to .4 range are good…some of these don’t reach that bar. 

 The discussion has too many unwarranted claims, for instance:

 “The full questionnaire also accounted for a substantial proportion of the variance in symptoms of anxiety and depression across both data sets.”  That’s not really true for most of the results, suggesting social media dimensions account for only a small percentage of the variance, assuming my understanding of “median” is correct.

 Avoid lofty claims like:: “help identify adolescents at risk of anxiety and depression related to social media use”  or “aimed at addressing potential links between social media use and mental health outcomes in adolescents” or “…has the potential to enhance understanding of mental health challenges associated with social media use, contributing to improved health outcomes for adolescents”

 Much of these statements as well as the discussion more generally involve causal implications regarding social media use that can’t be supported by the current data.

Is the work clearly and accurately presented and does it cite the current literature?

Partly

If applicable, is the statistical analysis and its interpretation appropriate?

Partly

Are all the source data underlying the results available to ensure full reproducibility?

No

Is the study design appropriate and is the work technically sound?

No

Are the conclusions drawn adequately supported by the results?

No

Are sufficient details of methods and analysis provided to allow replication by others?

Partly

Reviewer Expertise:

Youth and technology including social media

I confirm that I have read this submission and believe that I have an appropriate level of expertise to state that I do not consider it to be of an acceptable scientific standard, for reasons outlined above.

## References

[1] : There is no evidence that time spent on social media is correlated with adolescent mental health problems: Findings from a meta-analysis. *Professional Psychology: Research and Practice* .2025;56(1) : 10.1037/pro0000589 73-83 10.1037/pro0000589

# Response

**Published:** 2025-07-01

**Authors:** Jens Christoffer Skogen

**Affiliations:** Department of Health Promotion, Norwegian Institute of Public Health, Bergen, Vestland Fylkeskommune, Norway

Reviewer #1: Christopher Ferguson; **Not approved.**

 I appreciated the authors efforts to develop a scale that better assesses youth social media use.  However, I have some observations which reduce my enthusiasm.

 First, the authors need to better balance their lit review, particularly they need to cite null studies related to social media use and mental health to make clear time spent is a poor predictor (see for example, recent meta-analyses of Ferguson, Kaye, Branley-Bell & Markey, in press with Professional Psychology: Research and Practice (2025 [Ref-1]) or Yang and Feng, 2024 in Heliyon). 

**Response: We thank the reviewer for this suggestion. We agree that we could have been clearer when presenting the overall findings of “time spent” as a predictor of mental health outcomes. We have now added additional text regarding this in the first paragraph of the revised manuscript. This includes:**

***“In the last 15 years, a large volume of research has been focused on the potential link between social media use and mental health outcomes. Initially, the primary focus was on the amount of time used on social media, but currently a more fine-grained and purposeful focus has been advocated. This reflects the growing awareness that the total time spent on social media is a crude and imprecise measure for a host of different exposures and interactions. Specifically, time spent on social media has been described as an overly simplistic metric, analogous to measuring 'time spent' at school or home, when assessing risk factors for mental health problems. Importantly, several reviews of the literature have concluded that it is a poor predictor for mental health outcomes.”***

 I believe the authors overstate how “consistently related” specific subdomains are as well.  For instance, the concept of problematic social media use remains highly controversial and citing it as beyond controversy reduces my confidence in the authors’ objectivity.

**Response: We agree that measures and conceptualizations of other aspects of social media use also have it challenges, perhaps especially for “problematic social media use” and/or “social media addiction”. We do, however, believe that such an approach holds more promise than focusing on time spent on social media (whether is self-report or not log-based). We have tried to nuance the presentation of this section a little more in the first paragraph of the revised manuscript in order to also show the potential challenges with these other measures. Specifically, we have added the following:**

***“These other, more nuanced, measures of social media use are not without their own issues, however. The conceptualisation of problematic social media use, have for instance been based on several different theoretical frameworks (usually with behavioural addiction as a vantage point) and lack of focus on underlying mechanisms, making it difficult to compare results across studies, and challenging legitimacy of findings. Despite challenges in conceptualization, more specific measures of social media use remain a more promising research avenue than mere simple time-based metrics.***

***In line with this effort to move beyond time-based metrics, several different scales have been developed in recent years. These scales typically focus on more specific aspects of social media use, such as digital stress, problematic social media use, social media engagement, digital habits, passive-or-active use, perceived social support or feelings about social media. The diversity of these scales reflects the complex, multifaceted nature of social media as a phenomenon, but also introduces challenges for research synthesis and practical application.”***

 One issue I worried about was the fairly obvious demand characteristics of the current study which could create false correlations and unwarranted confidence in the survey.  For future studies, including distractor questionnaires may help.

 Some items did not impress me as helpful and may result in overpathologizing social media use (e.g. “I spend a lot of time and energy on what I post on social media”, even “I spend too much time on social media” which many teens may simply because they are constantly told this by parents, teachers, news media, etc., so they think it’s the correct response).  Others such as “My parents/guardians think I spend too much time on social media”…every parent has thought this about every form of media for centuries…it’s just not diagnostic. 

 It occurs to me that single-responder bias may also be a source of elevated correlations. 

**Response: We agree that we should have made these potential sources of bias (demand characteristics, single-responder bias, overpathologizing) more explicit in the manuscript. In the revised manuscript we now acknowledge the potential for bias – although we believe that the framing of the study may have alleviated some of the demand characteristics as the invitation letter emphasised the exploratory nature of the study and explicitly focused on potential positive and negative aspects of social media use. Addition to limitations:**

**“Fourthly, the data collection relies on self-report and is based on a single informant (the adolescents). This may lead to single-responder bias, increasing the risk that observed associations reflect individual reporting tendencies rather than true relationships. It also makes our data collection vulnerable to common method bias which may have inflated or distorted observed associations between variables. Relatedly, demand characteristics may also be a concern. While the explicit description of the study’s aims in the invitation letter introduces the potential for demand characteristics—where participants may tailor their responses to align with perceived expectations—framing the project as an exploration of both positive and negative aspects of social media use likely reduced this specific bias. By signalling an openness to a full spectrum of experiences, rather than reinforcing the perhaps more common negative narrative, the invitation may have encouraged more balanced and authentic responses from participants, thereby mitigating some of the limitations typically associated with demand characteristics.”**

**We have also added a little more information about the framing of the study in the methods section:**

***“The invitation letter to both the pilot and the “LifeOnSoMe”-study described the project as an initiative to better understand social media as a social arena for adolescents, which could hold benefits as well as challenges. It also explicitly informed participants that the study aimed to explore both positive and negative associations between social media use and young people’s mental health and well-being.”***

**The items included are based on focus group interviews of adolescents, and we believe that they hold experiential relevance in relation to adolescents’ views, experiences and motivations to use social media. Specifically, we think this approach supports the notion that the items are grounded in the lived experiences, perspectives, and motivations even if they are indeed also influenced by the framing and purpose of our study, parental and societal views of social media and peer influence.**

 In the regression tables, is “median” the median correlation?  I apologize if I missed that explanation somewhere.  I just wasn’t quite sure what “median” referred to.

**Response: We see that we have been unclear about this in the original manuscript. We have now revised and expanded the description of the statistical analyses and changed the labels of the columns in table 6. Specifically, we now make it clear that this column gives the results of the median posterior estimates, and that they express the average change in the dependent variable as standard deviation for each increase in the original scale of the independent variable. The mean posterior estimates were virtually identical to the median posterior estimates.**

 Some validity coefficients are on lower end.  Usually validity correlations in the .3 to .4 range are good…some of these don’t reach that bar. 

**Response: We are not sure what the reviewer means by validity coefficients but if it refers to the median regression coefficients in table 6, we agree that some of them should be considered a very small effect size, especially for “subjective overuse” in the model development dataset. We have now expanded on the results section and the first part of the discussion section to highlight the differences in the strength of association for the different domains.**

 The discussion has too many unwarranted claims, for instance:

 “The full questionnaire also accounted for a substantial proportion of the variance in symptoms of anxiety and depression across both data sets.”  That’s not really true for most of the results, suggesting social media dimensions account for only a small percentage of the variance, assuming my understanding of “median” is correct.

**Response: We agree the reviewers’ comments about this, and the interpretation of the explained variance has now been removed from the revised discussion and from the abstract.**

 Avoid lofty claims like:: “help identify adolescents at risk of anxiety and depression related to social media use”  or “aimed at addressing potential links between social media use and mental health outcomes in adolescents” or “…has the potential to enhance understanding of mental health challenges associated with social media use, contributing to improved health outcomes for adolescents” Much of these statements as well as the discussion more generally involve causal implications regarding social media use that can’t be supported by the current data.

**Response:** **We agree and see that we have been overzealous in this section of the manuscript. In the revised version, this section has been extensively shortened and focus on the scale’s potential usefulness in research.**
